# Clinical predictors of outcome in patients with inflammatory dilated cardiomyopathy

**DOI:** 10.1371/journal.pone.0188491

**Published:** 2017-12-21

**Authors:** Konstantinos Karatolios, Volker Holzendorf, George Hatzis, Dimitrios Tousoulis, Anette Richter, Bernhard Schieffer, Sabine Pankuweit

**Affiliations:** 1 Department of Cardiology, Angiology and Intensive Care, Philipps University Marburg, Marburg, Germany; 2 Clinical Trial Center Leipzig, Faculty of Medicine, University of Leipzig, Leipzig, Germany; 3 Department of Cardiology, Hippokration General Hospital, Athens University Medical School, Athens, Greece; Universitatsklinikum Wurzburg, GERMANY

## Abstract

**Objectives:**

The study objectives were to identify predictors of outcome in patients with inflammatory dilated cardiomyopathy (DCMi).

**Methods:**

From 2004 to 2008, 55 patients with biopsy-proven DCMi were identified and followed up for 58.2±19.8 months. Predictors of outcome were identified in a multivariable analysis with a Cox proportional hazards analysis. The primary endpoint was a composite of death, heart transplantation and hospitalization for heart failure or ventricular arrhythmias.

**Results:**

For the primary endpoint, a QTc interval >440msec (HR 2.84; 95% CI 1.03–7.87; p = 0.044), a glomerular filtration rate (GFR) <60ml/min/1.73m^2^ (HR 3.19; 95% CI 1.35–7.51; p = 0.008) and worsening of NYHA classification during follow-up (HR 2.48; 95% CI 1.01–6.10; p = 0.048) were univariate predictors, whereas left ventricular ejection fraction at baseline, NYHA class at entry, atrial fibrillation, treatment with digitalis or viral genome detection were not significantly related to outcome. After multivariable analysis, a GFR <60ml/min/1.73m^2^ (HR 3.04; 95% CI 1.21–7.66; p = 0.018) remained a predictor of adverse outcome.

**Conclusions:**

In patients with DCMi, a prolonged QTc interval >440msec, a GFR<60ml/min/1.73m^2^ and worsening of NYHA classification during follow-up were univariate predictors of adverse prognosis. In contrast, NYHA classification at baseline, left ventricular ejection fraction, atrial fibrillation, treatment with digitalis or viral genome detection were not related to outcome. After multivariable analysis, a GFR <60ml/min/1.73m^2^ remained independently associated with adverse outcome.

## Introduction

Inflammatory cardiomyopathy is defined as inflammation of the heart muscle associated with impaired function of the myocardium [1]. Myocarditis is defined as inflammation of the heart muscle and can lead to dilated cardiomyopathy in up to 30% of patients [1,2]. Inflammatory dilated cardiomyopathy (DCMi) is also characterized by inflammation of the heart muscle in addition to dilation and impaired contraction of the left or both ventricles that is not explained by abnormal loading conditions or coronary artery disease. In patients with initially unexplained heart failure a prevalence of 9–10% for inflammatory cardiomyopathy as underlying cause was reported [3,4]. For diagnosis endomyocardial biopsy (EMB) is crucial since confirmation of diagnosis is based on immunohistochemical evidence of myocardial inflammation. DCMi is considered to be a major cause of dilated cardiomyopathy (DCM) [5] and also one of the most frequent causes of sudden cardiac death, especially in younger patients [1,6]. Dilated cardiomyopathy in turn is the most common diagnosis leading to heart transplantation [7]. In patients with suspected myocarditis undergoing EMB positive immunohistology for infiltrating immune cells and expression of HLA-DR-a molecules, but not the classical histological Dallas criteria or viral genome detection were shown to be predictors of poor outcome [8]. However, other investigations including these methods yielded controversial results in patients with inflammatory cardiomyopathy [9,10]. Therefore, long-term prognosis of DCMi still remains a matter of debate suggesting that additional, preferably non-invasive, clinical markers are needed to assess the clinical course and to better identify patients at increased risk for adverse events. To our knowledge, only few studies [8,11] investigated clinical predictors of outcome in inflammatory cardiomyopathy. However, these studies included patients with myocarditis and inflammatory cardiomyopathy and not exclusively patients with DCMi. Hence, since there are no studies that specifically focused on risk factors for DCMi, the prognostic value of clinical parameters in DCMi remains elusive. In our previous study in patients with non-ischemic dilated cardiomyopathy [12] (including the subgroup of patients with DCMi), we identified a reduced systolic left ventricular ejection fraction (LVEF) < 35%, a prolonged QTc interval >440msec and an abnormal renal function with a glomerular filtration rate (GFR) <60ml/min/1.73m^2^as independent predictors of death or need for heart transplantation. Aiming to study specifically risk factors in DCMi, we investigated in the present study the potential of such clinical parameters as predictors of death, heart transplantation and hospitalization for heart failure or ventricular arrhythmias in this subgroup of patients.

## Materials and methods

### Patients

From September 2004 to March 2008, we prospectively enrolled 272 consecutive patients with non-ischemic DCM. Of the entire cohort of 272 patients, who all underwent endomyocardial biopsy, a subgroup of 55 (20%) patients had biopsy-proven DCMi and were included in the present analysis. Patients between 18 and 75 years of age were included if they had a left ventricular ejection fraction of <45% and a Henry index >117% estimated by echocardiography with no evidence of significant valve disease. Coronary artery disease (>50% diameter luminal stenosis in one or more epicardial vessels) was excluded in all patients by means of coronary angiography.

All patients underwent a careful history and clinical examination as well as laboratory studies and echocardiographic assessment with 2-dimensional echocardiography. Measurement of variables was based on the harmonized assessment protocol for patients with DCMi used within the Competence Network Heart Failure Germany. The diagnosis of DCM was made according to criteria of the position statement from the European Society of Cardiology working group on myocardial and pericardial diseases [13,14]. The diagnosis of myocardial inflammation was established if ≥ 14 leucocytes/mm^2^(including ≥ 7cells/mm^2^ CD3 positive T-lymphocytes and CD68-positive macrophages) were detected [1]. Patients were excluded from the study if they demonstrated one or more of the following parameters: peripartum cardiomyopathy, history of myocardial infarction, systemic hypertension, alcohol abuse, drug dependency.

The study was approved by the local institutional ethics committee and all patients provided written informed consent.

### Analysis of endomyocardial biopsies

At least 4 biopsy samples from each patient were obtained and processed. All biopsies were taken from the left ventricle. Analysis of EMBs included conventional histology, immunohistochemistry and molecular biology for the detection of cardiotropic viruses and was performed as described previously [1,15]. In brief, immunohistochemistry was performed to demonstrate infiltrating cells by antibodies specific for activated T and B cells, macrophages, major histocompatibility class 1 and class 2 antigens, adhesion molecules and endothelial cells. Specific binding of the antibodies indicating an inflammatory reaction was demonstrated by peroxidase double staining procedure. Inflammation in endomyocardial biopsies was diagnosed by the presence of ≥ 14 leucocytes/mm^2^. For detection of cardiotropic viruses in the EMBs the QIAamp Tissue Kit (Qiagen, Hilden, Germany) was used to extract total DNA and RNA from the biopsy samples. Primer pairs specific for coxsackievirus B, parvovirus B19, cytomegalovirus, adenovirus type 2, influenza virus A, herpes simplex virus, human herpesvirus 6 and Epstein–Barr virus were used to perform polymerase chain reaction and in case of PVB19 quantitative real-time polymerase chain reaction. Polymerase chain reaction results were confirmed by southern blot hybridization.

### Study design, follow-up and end points

The study was designed as a prospective observational investigation. Follow-up visits included clinical examination, a 12-lead ECG, laboratory studies and a transthoracic echocardiography. Glomerular filtration rate (GFR), expressed as ml/min/1.73m^2^, was computed using the formula derived from the Modification of Diet in Renal Disease (MDRD) study [16]. QTc intervals were calculated using Bazett’s formula. A prolonged QTc interval was defined as an interval >440msec. Patients with bundle branch block or permanent pacing at baseline were not included in the QTc analysis. Improvement in LVEF was defined as LVEF increase within one year by at least 10%-points compared to baseline. The echocardiography had to be separated at least 6 months from baseline echocardiography. The primary end point was a composite of death, heart transplantation and hospitalization for heart failure or ventricular arrhythmias (sustained ventricular tachycardia or ventricular fibrillation).

### Statistical analysis

Subject’s characteristics are described by N (%) or mean ± standard deviation (SD) as appropriate. The first analytic step was an unadjusted analysis for each parameter and afterwards adjusted for age. In a next step, we included all univariate significant parameters in one model to identify the independent impact of each parameter. To determine the effect of the identified risk factors we analyzed the data with Cox proportional hazard models. Time to first occurrence of the defined endpoint was analyzed by calculating hazards ratios (HR) and corresponding 95% confidence intervals.

Analyses were performed with R version 3.1.2 (R Core Team).

## Results

### Patient population

The baseline characteristics of our study patients are shown in Table 1. The mean age of patients was 51.1±11.6 years and 76% were men. Severely reduced LVEF (≤35%) was present in 42 (76.4%) patients and 50% of the patients (N = 28) had severe heart failure symptoms (NYHA III and IV).

**Table 1:** Baseline characteristics of study patients (N = 55).

Characteristic	Value
Age (years)	51.1 ± 11.6
BMI (kg/m^2^)	27.9±4.2
Female, n (%)	13 (24)
SBP (mmHg)	118±17
DBP (mmHg)	76±11
NYHA functional class, n (%)	
• I	4 (7)
• II	23 (42)
• III	25 (45)
• IV	3 (5)
NYHA functional class during follow up, n(%)*	
• Improvement	22 (40)
• Worsening	6 (11)
QTc time (Bazett) (ms)	449±50
Duration of heart failure symptoms < 6 months^§^	21 (38)
LVEDD (mm) at baseline	70.1±9.2
LVEDD (mm) during follow up*	65.3±12.1
LVEF (%)	29.2±8.5
Mitral regurgitation at baseline	
• none	21(38)
• mild	27(49)
• moderate	7(13)
Mitral regurgitation during follow-up*	
• improvement	11 (20)
• worsening	6 (11)
LVEDP (mmHg)	19.2±8.8
Mean PAP (mmHg)	23.0±9.2
Medication	
• ACEI or ARB	52 (95)
• Betablocker	47 (85)
• Mineralocorticoid receptor antagonists	36 (65)
• Digitalis	34 (62)
• Diuretic	48 (87)
Creatinine (mg/dl)	1.05±0.32
GFR (ml/min/1.73m^2^)	82.0±26.7
ICD	
• At baseline	23 (42)
• Implanted during follow-up	10 (18)
Results of EMB	
Number of leukocytes/mm^2^	23.6±37.2
Myocardial fibrosis (histopathological)	
• None	11 (20)
• Mild	26 (47)
• Moderate	10 (18)
• Severe	8 (15)
Virus-positive EMBs	16 (29)
• PVB19	14
• CMV	1
• HSV	1

Values are n (%) or mean ± SD when appropriate. BMI: body mass index, NYHA: New York Heart Association, SBP: systolic blood pressure, DBP: diastolic blood pressure, LVEF: left ventricular ejection fraction, LVEDD: left ventricular enddiastolic diameter, LVEDP: left ventricular enddiastolic pressure, PAP: pulmonary artery pressure, ACEI: angiotensin converting enzyme inhibitor, ARB: angiotensin receptor blocker, ICD: intracardiac cardioverter defibrillator, GFR: glomerular filtration rate, EMB: endomyocardial biopsy, PVB19: parvovirus B19, CMV: cytomegalovirus, HSV: herpes simplex virus.

* within 1 year of follow-up;

^§^ duration of symptoms before study inclusion.

Heart failure treatment with angiotensin-converting enzyme inhibitors (ACEI) or angiotensin-receptor blocker (ARB), β-blockers, mineralocorticoid receptor antagonists (MRA) and glycosides were given to 95% (N = 52), 85% (N = 47), 65% (N = 36) and 62% (N = 34) of patients, respectively (Table 1). ACEI/ARB or β-blockers were not included in the analysis, because of the low prevalence of patients not treated with these medications (N = 3, 5% for ACEI/ARB and N = 8, 15% for β-blockers).

Of the 55 patients with DCMi, 16 (29%) patients were positive for viral genome detected in EMB analysis. Parvovirus B19 was found in 14, herpes simplex virus in 1 and cytomegalovirus in 1 patient.

Immunosuppressive treatment was given to 9 (16%) patients (all negative for viral genome), whereas all patients with detection of viral genome were treated with intravenous immunoglobulins.

After the first year of follow-up, systolic LVEF improved in 24 (44%) patients (Table 2). There were no significant differences between patients with or without improvement of LVEF including age, gender, LVEF at baseline, NYHA functional class at entry, QTc interval, presence of mitral regurgitation, heart failure treatment or EMB results (including inflammatory cell count and myocardial fibrosis). However, renal dysfunction was significantly more prevalent in patients without improvement of LVEF (Table 2). Worsening of NYHA functional class was observed in 6 (11%) and improvement in 22 (40%) patients during the first year of follow-up. Increase in mitral regurgitation during the first year of follow-up was observed in 6 (11%) and improvement in 11 (20%) patients. However, no patient deteriorated to severe mitral regurgitation.

**Table 2:** Characteristics of patients with or without LVEF improvement*.

Characteristic	LVEF improvement (N = 24)	No LVEF improvement (N = 28)	p-value
Age (years)	48.6±11.4	53.4±12.0	0.15
BMI (kg/m^2^)	27.3±5.1	28.1±2.9	0.49
Female, n (%)	6 (25)	5 (18)	0.73
SBP (mmHg)	118±18	119±16	0.84
DBP (mmHg)	75±9	77±12	0.48
NYHA functional class			0.10
• I	3 (12)	1 (4)	
• II	6 (25)	17 (61)	
• III	12 (50)	10 (36)	
• IV	3 (12)	0	
QTc interval (msec)	444±52	451±49	0.60
LVEDD (mm)	69.9±8.1	70.8±10.4	0.74
LVEF (%) at baseline	27.3±9.6	47.0±7.5	0.07
Mitral regurgitation			0.51
• none	11 (46)	10 (36)	
• mild	10 (42)	14 (50)	
• moderate	3 (12)	4 (14)	
LVEDP (mmHg)	18.7±9.6	19.4±8.7	0.80
Mean PAP (mmHg)	23.2±8.8	22.7±10.4	0.86
Treatment			
• ACEI or ARB	22 (92)	27 (96)	0.59
• Betablocker	18 (75)	26 (93)	0.12
• Mineralocorticoid receptor antagonists	17 (71)	18 (64)	0.77
• Digitalis	17 (71)	17 (61)	0.56
• Diuretic	21 (88)	24 (86)	1.00
Creatinine (mg/dl)	0.95±0.25	1.18±0.35	**0.010**
GFR (ml/min/1.73m^2^)	90.1±23.0	72.9±26.6	**0.018**
Results of EMB			
Number of leukocytes/mm^2^	19.5±13.6	28.2±50.6	0.42
Myocardial fibrosis (histopathological)			0.76
• None	5 (21)	5 (18)	
• Mild	12 (50)	14 (50)	
• Moderate	4 (17)	5 (18)	
• Severe	3 (12.5)	4 (14)	
Viral genome, n	7 (29)	9 (32)	1.0

Values are n (%) or mean ± SD when appropriate. BMI: body mass index, NYHA: New York Heart Association, SBP: systolic blood pressure, DBP: diastolic blood pressure, LVEF: left ventricular ejection fraction, LVEDD: left ventricular enddiastolic diameter, LVEDP: left ventricular enddiastolic pressure, PAP: pulmonary artery pressure, ACEI: angiotensin converting enzyme inhibitor, ARB: angiotensin receptor blocker, GFR: glomerular filtration rate, EMB: endomyocardial biopsy.

* Complete echocardiographic evaluation (including LVEDD, LVEF, presence of mitral regurgitation) during follow-up was available in 52 (95%) of patients.

### Primary endpoint and predictors of outcome

**Fig 1:**
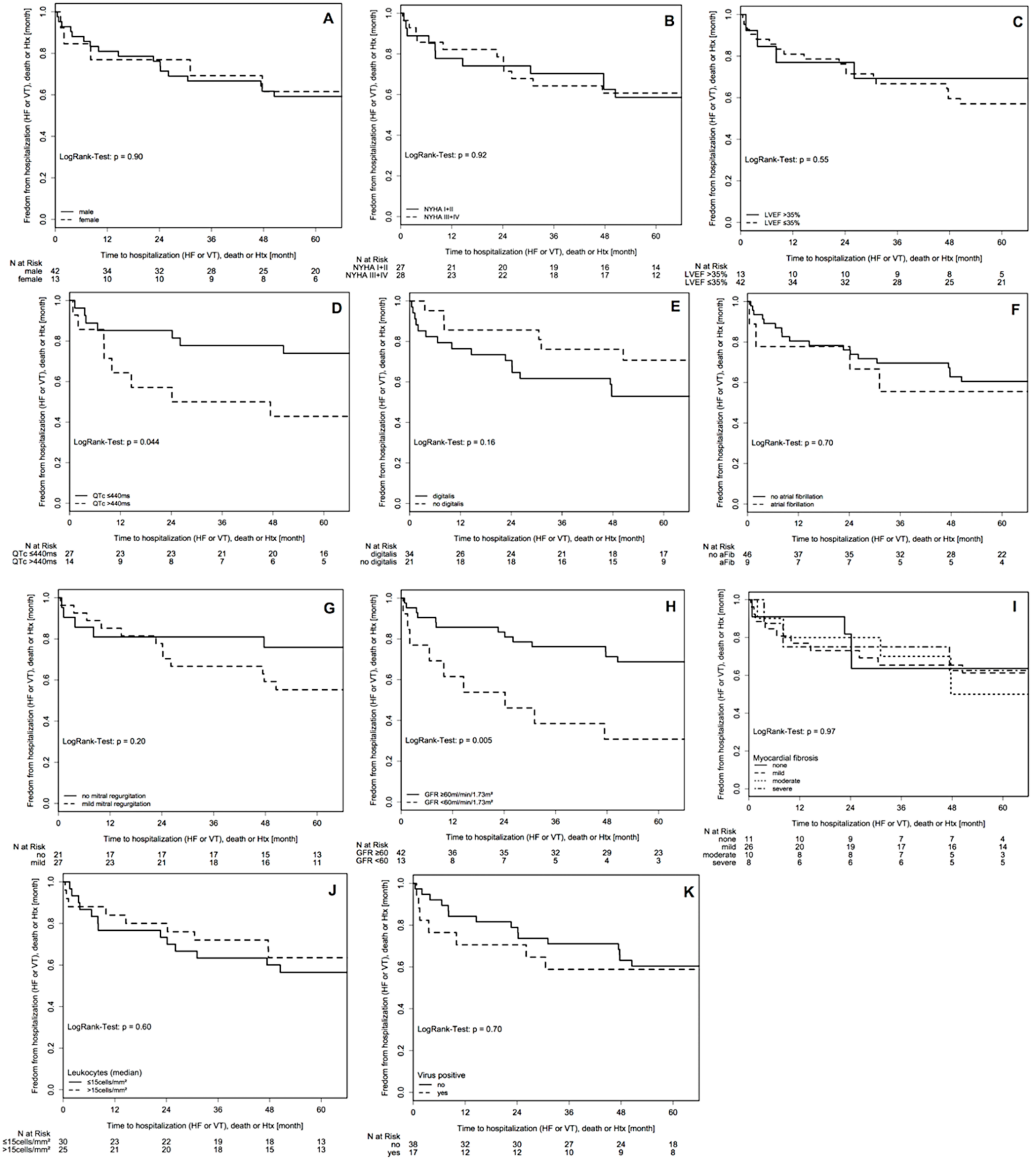
(a-k). Unadjusted survival free from death, heart transplantation and hospitalization for heart failure or ventricular arrhythmias in relation to clinical, laboratory, electrocardiographic, echocardiographic parameters and immunohistochemical parameters. a: gender; b: NYHA functional class; c: left ventricular ejection fraction (LVEF); d: QTc interval; e: treatment with digitalis; f: atrial fibrillation; g: mitral regurgitation; h: glomerular filtration rate (GFR), i: myocardial fibrosis, j: inflammatory cell count on endomyocardial biopsy, k: viral genome detection.

During the follow-up period (mean follow-up 58.2±19.8 months), 14 (25%) patients were hospitalized for heart failure or ventricular arrhythmias (10 for heart failure and 4 patients for ventricular arrhythmias). Of them, 9 patients died later on during follow-up. In 7 (13%) patients death was the first adverse event. Overall, 16 (29.1%) patients died during the study period. One patient (1.8%) underwent heart transplantation for end-stage heart failure. In total, 22 patients (40%) reached the primary endpoint. For the primary end point of all-cause mortality, heart transplantation and hospitalization for heart failure or ventricular arrhythmias a prolonged QTc interval >440msec, a GFR <60ml/min/1.73m^2^ and worsening of NYHA functional classification during follow-up were significant predictors in the univariate analysis, whereas gender, NYHA functional classification at entry, atrial fibrillation, systolic LVEF at entry, decrease of LVEDD at follow-up, mild mitral regurgitation, increase in mitral regurgitation during follow-up, treatment with digitalis, myocardial fibrosis, inflammatory cell count or viral genome detection in EMB were not significantly related to the endpoint. Fig 1(a)–1(k) shows the event-free survival of the study population in relation to clinical, laboratory, electrocardiographic, echocardiographic and immunohistochemical parameters as unadjusted Kaplan-Meier curves.

After multivariable analysis, a GFR <60ml/min/1.73m^2^ remained a significant predictor of the primary endpoint (Table 3). Specific treatment with immunosuppression or immunoglobulins had no influence on clinical outcomes.

**Table 3:** Hazard ratio for the composite end-point all-cause mortality, heart transplantation and hospitalization for heart failure or ventricular arrhythmias.

	Univariate analysis		Multivariable analysis	
	HR (95% CI)	p-value	HR (95% CI)	p-value
Gender	0.94 (0.35–2.54)	0.90		
NYHA class III/IV at baseline	0.96 (0.42–2.21)	0.92		
Worsening NYHA class*	2.48 (1.01–6.10)	0.048	2.12 (0.82–5.52)	0.12
Atrial fibrillation	1.24 (0.42–3.67)	0.70		
QTc >440 (msec)	2.84 (1.03–7.87)	0.044	1.65 (0.53–5.08)	0.38
LVEF <35%	1.39 (0.47–4.11)	0.55		
LVEDD decrease by 5mm*	0.46 (0.16–1.30)	0.14		
Mitral regurgitation (mild)	1.94 (0.68–5.52)	0.21		
Increase in mitral regurgitation*	1.67 (0.48–5.80)	0.42		
GFR <60ml/min/1.73m^2^	3.19 (1.35–7.51)	0.008	3.04 (1.21–7.66)	0.018
Digitalis	0.85 (0.67–1.07)	0.17		
Myocardial fibrosis				
• Mild	1.09 (0.34–3.49)	0.88		
• Moderate	1.34 (0.36–4.98)	0.67		
• Severe	1.02 (0.23–4.58)	0.98		
Leukocytes ≤15/mm^2^	0.80 (0.34–1.87)	0.61		
Detection of viral genome	1.19 (0.49–2.93)	0.70		

HR: hazard ratio, NYHA: New York Heart Association, LVEF: left ventricular ejection fraction, GFR: glomerular filtration rate. Only variables with a univariate value of p<0.05 were allowed to enter the multivariable analysis.

*within 1 year of follow-up.

## Discussion

The purpose of the present study was to investigate the long-term outcome and clinical predictors of outcome in patients with DCMi. Patients with DCMi had a 5-year mortality of 29.1% in our study. We observed that a prolonged QTc interval >440msec, a GFR<60ml/min/1.73m^2^ and worsening of NYHA functional classification during follow-up were univariate predictors of the composite endpoint of death, heart transplantation and hospitalization for heart failure or ventricular arrhythmias. In contrast, gender, NYHA functional classification at entry, atrial fibrillation, systolic LVEF at entry, decrease of LVEDD during follow-up, mild mitral regurgitation, increase in mitral regurgitation during follow-up, treatment with digitalis, myocardial fibrosis, inflammatory cell count or viral genome detection in EMB were not related to outcome. After multivariable analysis, only a GFR<60ml/min/1.73m^2^ remained a significant independent predictor of the primary endpoint.

LVEF is an accepted predictor of prognosis in patients with heart failure [17,18] and low LVEF is associated with poor prognosis [19,20]. However, our results showed that in patients with DCMi impaired LV function at entry was not significantly associated with adverse outcome. This may be due to the fact that in inflammatory cardiomyopathy impaired LV function is often reversible [21]. The basis for improvement of LV function seems to be related to the retreat of myocardial inflammatory infiltration and arrested production of negatively inotropic inflammatory mediators [2,22]. Along those lines, in our study LV systolic function improved in 24 (44%) patients within the first year of follow-up.

We observed that a reduced GFR<60ml/min/1.73m^2^ was also a predictor of the composite end point of death, need for heart transplantation and hospitalization for heart failure or ventricular arrhythmias in univariate and multivariable analysis. Decreased renal function is an strong and independent risk factor for adverse cardiovascular outcomes in a broad spectrum of patients with heart failure, including those with reduced as well as preserved LVEF [23,24]. In patients with ischemic and non-ischemic DCM, renal dysfunction was also associated with adverse cardiac events [25,26].

Most investigations on the prognostic relevance of QTc interval in heart failure included patients with mixed etiologies of cardiomyopathy with ischemic and non-ischemic genesis [27,28], whereas we specifically focused on patients with DCMi. Moreover, the very few investigations on QTc interval in non-ischemic DCM [29] focused on patients with idiopathic DCM, so that, to our knowledge, there are no studies on QTc interval duration in pure DCMi patients. However, the study by Ukena et al [30] included patients with suspected myocarditis, of whom 51.5% had positive immunohistochemical signs of myocardial inflammation in EMB. In this study, prolonged QRS duration was an independent predictor for cardiac death and heart transplantation in patients with myocarditis, whereas prolonged QTc interval was also associated with adverse clinical outcome, but only in univariate analysis. This finding is in line with the results of our study, as a prolonged QTc interval > 440msec had a negative effect on the prognosis in our patients’ with DCMi in univariate analysis. In contrast the study by Hombach et al [29] showed no prognostic significance of prolonged QTc interval in patients with idiopathic dilated cardiomyopathy. However, in this study patients with myocardial inflammation were excluded. It is therefore highly probable that mechanisms and incidence of adverse outcomes (such as ventricular arrhythmias and death) differ in patients with myocardial inflammation compared to patients with non-inflammatory cardiomyopathy.

The prognostic significance of atrial fibrillation in patients with heart failure remains inconclusive [31]. Many studies have failed to demonstrate an independent association of atrial fibrillation with mortality in heart failure patients [32–34], whereas a very recent meta-analysis suggested an adverse prognostic impact of atrial fibrillation in heart failure [35]. However, the investigations on the prognostic relevance of atrial fibrillation in heart failure included patients with mixed etiologies of heart failure. In our study in patients with DCMi, atrial fibrillation was not associated with adverse prognosis.

Digitalis has historically been one of the most commonly prescribed drugs in chronic heart failure with reduced LVEF, but whether digitalis offers clinical benefit in heart failure enough to compensate for its well-recognized risk of toxicity is not clarified. In the randomized Digitalis Investigation Group (DIG) trial [36] digitalis had a neutral effect on mortality, but it reduced the rate of hospitalization for worsening heart failure. However, the DIG trial predated the use of beta-blockers and MRAs for heart failure treatment [36] and therefore has a doubtful clinical application to the current clinical context. Furthermore, prospective trials, especially in patients with DCMi under contemporary heart failure therapy, including beta-blockers and MRAs, are lacking. In addition to that, animal models point towards a possible adverse effect of digitalis in patients with myocarditis or inflammatory cardiomyopathy [37]. In our study, treatment with digitalis added to angiotensin converting enzyme-inhibitors or angiotensin receptor blockers, beta-blockers and mineralocorticoid antagonists in patients with DCMi had no effect on outcome or hospitalization as compared to patients without digitalis.

Inflammatory cell count in EMB did not reach statistical significance for the primary endpoint. Similarly, detection of viral genome in EMB was not significantly associated with increased risk of death, heart transplantation or hospitalization for heart failure or ventricular arrhythmias. Current pathological evaluation of EMB focuses on detailed immunohistochemical analysis for identification and characterization of inflammatory infiltrates [1]. However, investigations including these methods yieldedso far controversial results in patients with inflammatory cardiomyopathy [8,9,11,38]. The investigation by Kindermann et al. [8] demonstrated that in patients with suspected myocarditis undergoing endomyocardial biopsy positive immunohistology for infiltrating immune cells and expression of HLA-DR-a molecules, but not viral genome detection, were independent predictors of poor outcome. On the other hand, in studies by Caforio et al. [11] and Kuehl et al. [38] viral genome detection at diagnosis of inflammatory cardiomyopathy and viral persistence are associated with adverse prognosis. Moreover, a recent investigation in patients with biopsy-proven myocarditis did not observe significant associations between outcome and immunohistochemical staining targets including CD3-positive T-lymphocytes or CD68-positive macrophages [9]. These controversial results on the prognostic value of immunohistochemical evaluation of EMB are in our view, at least in part, explained by the varying etiopathogenetic features of inflammatory cardiomyopathy [1] and the heterogeneities among the studied patients. However, these results suggest that additional clinical markers are needed for risk prediction in patients with biopsy-proven DCMi.

### Study limitations

Several limitations have to be acknowledged. First of all, given to the small number of patients enrolled in the study, it must be kept in mind that the number of events may still be too small to exclude moderate relations of some of the variables tested to outcome with certainty. Furthermore, the present study was not designed to evaluate the prognostic value of immunohistochemical analysis of inflammatory infiltrates in EMB. Therefore, we cannot exclude that the lack of significant association between inflammatory cell counts on EMB and outcome is due to the small number of patients studied, since the larger study by Kindermann et al. [8] proved the prognostic value of immunohistochemical evidence of inflammatory infiltrates on EMB in a larger cohort of patients. Regarding the treatment with digitalis, serum digitalis levels were not checked routinely, which most likely reflects clinical practice of assessing digitalis levels only in cases of suspected toxicity. However, we could not evaluate whether digitalis levels would have modified the effect on outcomes.

## Conclusions

We were able to demonstrate that a prolonged QTc interval >440msec, an abnormal renal function with a GFR<60ml/min/1.73m^2^ and worsening of NYHA functional classification during follow-up were univariate predictors of adverse prognosis patients with DCMi, whereas gender, NYHA functional classification at entry, atrial fibrillation, systolic LVEF at entry, mild mitral regurgitation, treatment with digitalis or viral genome detection in EMB were not related to outcome. After multivariable analysis, only a GFR<60ml/min/1.73m^2^ remained a significant independent predictor of death, heart transplantation and hospitalization for heart failure or ventricular arrhythmias.

## Supporting information

S1 Data set This is the data set file. (XLSX)
